# How bacteria arrange their organelles

**DOI:** 10.7554/eLife.43777

**Published:** 2019-01-10

**Authors:** Emilia Mauriello

**Affiliations:** Laboratoire de Chemie BactérienneCentre National de la Recherché ScientifiqueMarseilleFrance

**Keywords:** *Synechococcus elongatus* PCC 7942, cyanobacteria, carboxysomes, protein self-organization, Other

## Abstract

The structures responsible for photosynthesis in bacteria use the nucleoid and two unique proteins as a scaffold to position themselves.

**Related research article** MacCready JS, Hakim P, Young EJ, Hu L, Liu J, Osteryoung KW, Vecchiarelli AG, Ducat DC. 2018. Protein gradients on the nucleoid position the carbon-fixing organelles of cyanobacteria. *eLife* **7**:e39723. doi: 10.7554/eLife.39723

The emergence of cell biology techniques proved that bacterial cells are compartmentalized. Indeed, many of the activities performed inside bacterial cells are restricted to specific regions, like the cell wall, or to organelles (Murat et al., 2010; Mauriello et al., 2018). Photosynthetic cyanobacteria contain organelles called carboxysomes: specialized compartments that encapsulate the key enzymes for photosynthesis in a protein shell. In the cyanobacterium *Synechococcus elongatus*, carboxysomes align themselves at equal distances from each other along the longitudinal axis of the cell. This distribution means that each daughter of a dividing cell receives its fair share of carboxysomes and can photosynthesize soon after forming.

The amount of a protein called McdA oscillates inside bacteria, leading to regions that contain high levels of McdA and regions that contain low levels, and in 2010 researchers established a link between these oscillations and the positioning of carboxysomes within bacteria (Savage et al., 2010). However, it was not known what causes the McdA oscillations, or how these dynamics determine the arrangement of the carboxysomes. Now, in eLife, Daniel Ducat of Michigan State University, Anthony Vecchiarelli of the University of Michigan and co-workers – including Joshua MacCready as first author – report the molecular mechanism behind these processes in *S. elongatus* (MacCready et al., 2018).

Firstly, MacCready et al. showed that McdA oscillations take place on the nucleoid, the region within a bacterium that is occupied by DNA. Carboxysomes also localize at this position. The researchers then discovered a small protein that is able to interact directly with McdA and also with some of the proteins that make up the carboxysome shell. This protein, which MacCready et al. called McdB, thus acts as a bridge to connect the carboxysomes with McdA at the nucleoid.

But what causes the McdA oscillations? McdA binds to the nucleoid when bound to ATP, a molecule that releases energy when it is hydrolyzed. McdA is able to hydrolyze ATP highly efficiently, and this activity is further enhanced by McdB. Experiments in vitro and in vivo show that by promoting the ability of McdA to hydrolyze ATP, McdB helps McdA to detach from the nucleoid. This creates regions on the nucleoid that are depleted of McdA. Because McdB tends to localize at high concentrations of McdA, the carboxysomes move toward those regions of the nucleoid that are rich in McdA. The end result is that the carboxysomes become evenly spaced along the nucleoid. The McdA oscillations emerge from the presence of multiple McdB-containing carboxysomes, which cause McdA to repeatedly dissociate from and then re-associate with the nucleoid.

MacCready et al. performed an elegant experiment that explains and confirms the predictions of this model. Using different gene expression systems, they were able to produce cells that contained one, two or more carboxysomes. The nucleoid, carboxysomes and McdA inside these cells were fluorescently labeled to enable their behavior to be tracked using a microscope.

In cells with one carboxysome, the organelle localizes at the only McdA-depleted region of the nucleoid (Figure 1). In cells with two carboxysomes, the more central carboxysome moves away from the other one and toward the highest concentration of McdA. When they are sufficiently far apart, McdA reassembles on the McdA-depleted region of the nucleoid, and the more central carboxysome slightly moves back. In cells with multiple carboxysomes, the movements of the carboxysomes and the resulting McdA oscillations cause the organelles to space themselves equidistantly.

**Figure 1.**
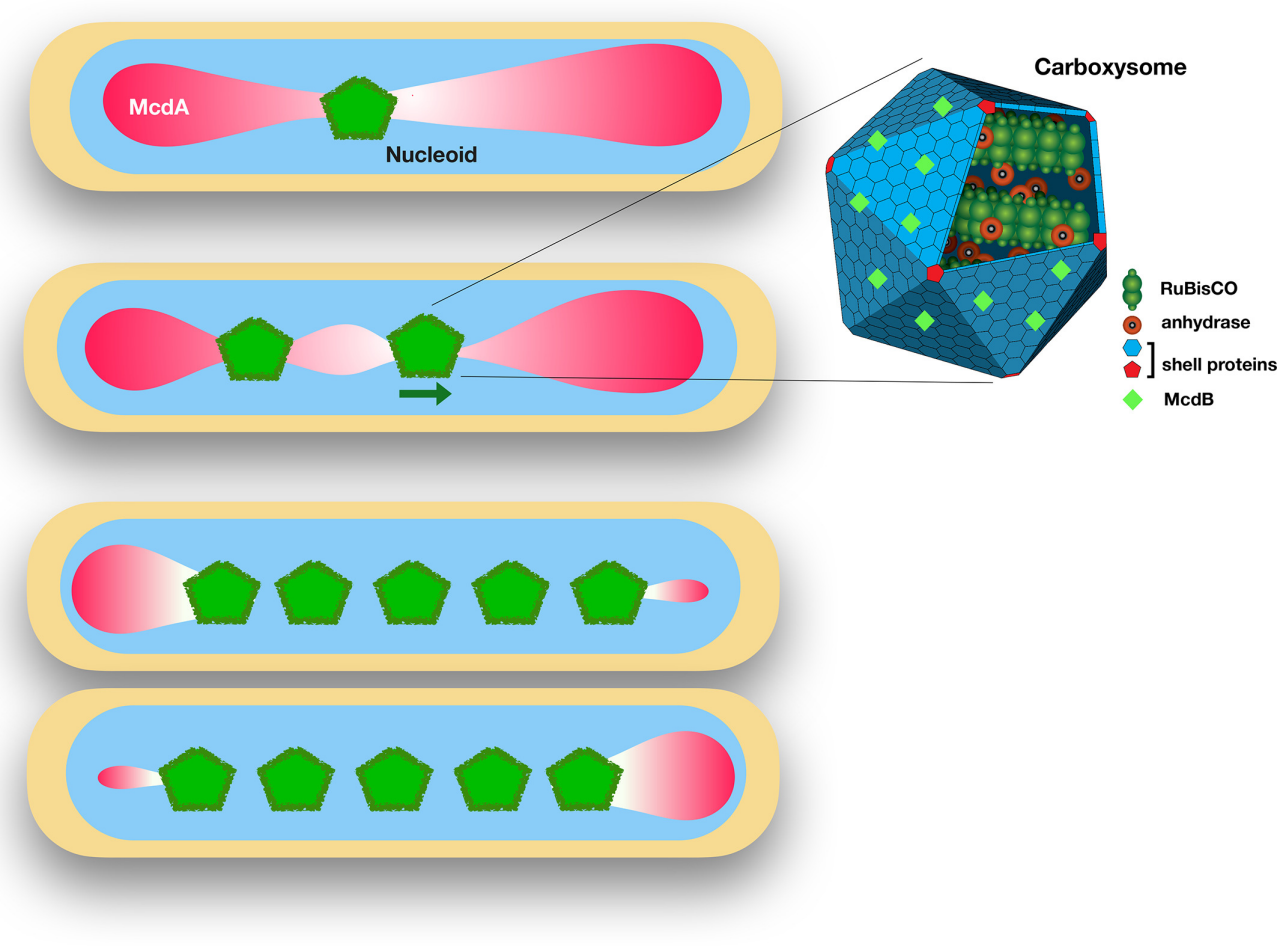
The proteins McdA and McdB interact to position carboxysomes in bacterial cells. Left: *Synechococcus elongatus* cells bearing one or multiple carboxysomes (green pentagons). McdB proteins on the surface of the carboxysomes create a gradient of McdA (pink) that oscillates across the surface of the nucleoid. Carboxysomes move to the highest concentration of McdA, but McdB causes McdA to dissociate more easily from the nucleoid surface. In cells containing one carboxysome (top), the carboxysome sits at the McdA-depleted region of the nucleoid. In cells containing two or more carboxysomes, the carboxysomes move apart from each other until they end up equally spaced across the nucleoid. Right: Schematic diagram of a carboxysome (adapted from http://2014.igem.org/Team:Bielefeld-CeBiTec/Project/CO2-fixation/Carboxysome; CC BY 3.0). Carboxysomes contain the key enzymes for photosynthesis – RuBisCO and carbonic anhydrase. McdB proteins (light green diamonds) on the surface of carboxysomes allow the carboxysomes to interact with McdA on the surface of nucleoids.

MacCready et al. observe that this model fits a Brownian ratchet model (in which random motion can be used to move a cargo in one direction). A similar model has been proposed for the ParA–ParB segregation system that partitions chromosomes and plasmids (Vecchiarelli et al., 2014; Hu et al., 2017). Indeed, McdA is a ParA-like protein.

It has long been known that the cytoskeleton and the cell wall are the main organizers of the contents of bacterial cells. However, it has become clear that the nucleoid also serves as a scaffold for assembling large complexes and organelles (Thompson et al., 2006; Henry and Crosson, 2013; Moine et al., 2017; MacCready et al., 2018). When these complexes and organelles have to occupy specific positions in a cell, how does the cell ensure that they are inherited equally by both daughter cells after division? The latest results from MacCready et al. on carboxysomes add another example to the list of structures that ParA–ParB-like systems can segregate during cell division (for other examples, see Thompson et al., 2006; Alvarado et al., 2017). The presence of a reliable segregation system is essential for the emergence of bacterial populations in which all the cells perform the same function.
